# ﻿Morphology of a novel ciliate, *Oxytrichachongqingica* sp. nov. (Ciliophora, Hypotrichia)

**DOI:** 10.3897/zookeys.1247.155543

**Published:** 2025-07-25

**Authors:** Haifeng Han, Xiaojin Xue, Zhisheng Zhang, Chen Shao

**Affiliations:** 1 Laboratory of Biodiversity and Evolution of Protozoa in Wetland, College of Life Sciences, Shaanxi Normal University, Xi’an 710119, China Shaanxi Normal University Xi’an China; 2 Key Laboratory of Eco-environments in Three Gorges Reservoir Region (Ministry of Education), School of Life Sciences, Southwest University, Chongqing 400715, China Southwest University Chongqing China

**Keywords:** New species, Oxytrichidae, SSU rDNA, taxonomy

## Abstract

A novel hypotrichous ciliate, *Oxytrichachongqingica***sp. nov.**, was identified in Chongqing, southwestern China. This species is distinguishable from congeners by various qualitative and quantitative characteristics, such as body size and shape, pattern of endoral and paroral, number of macronuclear nodules, presence and arrangement of cortical granules, as well as ventral and dorsal ciliature. Phylogenetic analyses based on small subunit ribosomal DNA (SSU rDNA) sequences indicate that *Oxytrichachongqingica***sp. nov.** forms a cluster with *O.nauplia*, *Allotrichidesantarcticus*, *O.paragranulifera*, *O.quadricirrata*, *Paroxytrichalongigranulosa*, and *P.ottowi*, albeit with weak or moderate nodal support (82 ML and 0.87 BI).

## ﻿Introduction

The genus *Oxytricha* Bory de Saint-Vincent in Lamouroux et al. 1824 is taxonomically intricate among ciliated protists in comprising about 50 species. Recent discoveries of new species, such as *O.acidotolerans*Weisse et al., 2013, *O.atypica*Fan et al., 2021, *O.buxai* Bharti & Kumar, 2023, *O.granuliferachiapasensis*Méndez‐Sánchez et al., 2018, *O.multilineata*Jin et al., 2022, *O.paragranulifera*Shao et al., 2014, *O.seokmoensis* Kim & Min, 2019, and *O.xianica*Wang et al., 2021, indicate a substantial species diversity within *Oxytricha* (Weisse et al. 2013; Shao et al. 2014; Méndez‐Sánchez et al. 2018; Kim and Min 2019; Fan et al. 2021; Wang et al. 2021; Jin et al. 2022; Bharti and Kumar 2023).

In the present study, the living morphology and infraciliature of a novel hypotrich, *Oxytrichachongqingica* sp. nov. is described. Furthermore, sequence of the small subunit ribosomal DNA (SSU rDNA) and phylogenetic trees were employed to assess its evolutionary placement.

## ﻿Materials and methods

### ﻿Sampling and cultivation

**Figure 1.**
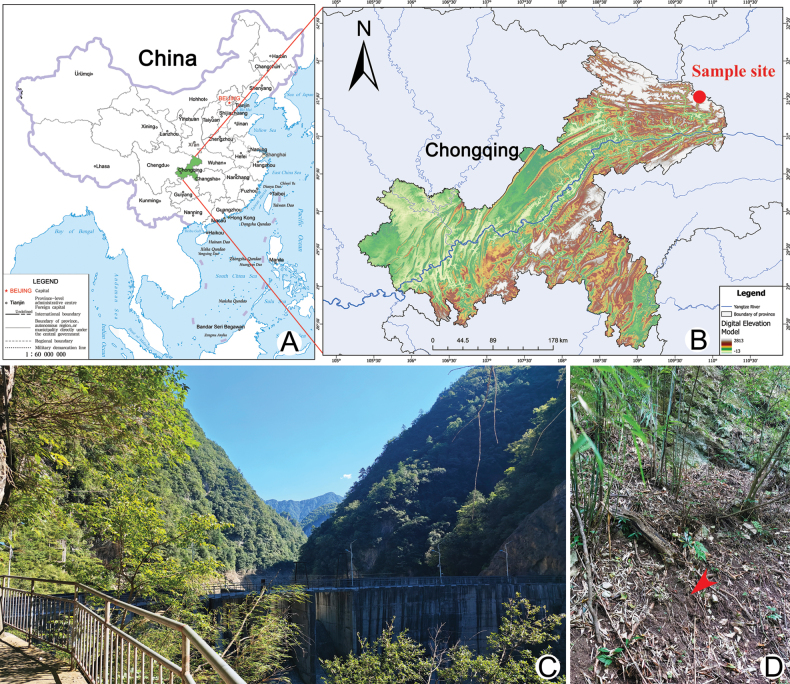
Location and surrounding of the sampling site. **A.** Location of Chongqing city in China (map from the National Platform for Common Geospatial Information Services (www.tianditu.gov.cn), Revision No. GS (2019) 1654); **B.** Location of the sample site; **C, D.** Surroundings at the sampling site; red arrowhead indicates where the samples were collected.

Soil (0–5 cm depth, including leaf litter and humus layers) samples containing *Oxytrichachongqingica* sp. nov. were collected on 13 August 2024 from Yintiaoling Nature Reserve (31°31'26"N, 109°49'31"E), Chongqing, southwestern China (Fig. 1A–D). Ciliates were induced to excyst following Foissner (1987) non-flooded Petri dish protocol. Cultures were maintained under ambient laboratory conditions (25 ± 1 °C) using mineral water (Nongfu Spring) with sterile rice grains to support bacterial growth as a nutritional source for the ciliates.

### ﻿Morphology

Live cell observations were conducted using an Olympus BX53 microscope equipped with differential interference contrast (DIC) optics and bright-field illumination, coupled with a DP74 digital imaging system for photomicrograph capture. The nuclear apparatus and infraciliature were examined through protargol staining (Sigma-Aldrich, cat. #448 K2787347) according to Wilbert (1975). Morphometric analyses of silver-impregnated specimens were performed under oil immersion (100× objective, 12.5× ocular). A depiction of a live specimen was generated based on photographic records, while stained cell morphologies were captured using a camera setup. The terminology used follows Berger (1999).

### ﻿DNA extraction, PCR amplification, and sequencing

Individual ciliates were aseptically isolated from culture media through three-stage purification with sterile distilled water (0.22 μm membrane filtration, Tianjin Branch Billion Lung Experimental Equipment Co., Ltd, China). Selected specimens were suspended in 1.5 mL microcentrifuge tubes containing minimal aqueous medium. Genomic DNA was isolated using the DNeasy Blood and Tissue Kit (Qiagen, Hilden, Germany) according to the manufacturer’s protocol. Amplification of SSU rDNA employed KOD OneTM PCR Master Mix-Blue-(TOYOBO, Cat# KMM-201) with universal eukaryotic primers (18S-F (5′-AAC CTG GTT GAT CCT GCC AGT-3′) and 18S-R (5′-TGA TCC TTC TGC AGG TTC ACC TAC-3′)) originally described by Medlin et al. (1988). Thermal cycling parameters comprised: initial denaturation at 98 °C (30 s); 18 touchdown cycles of 98 °C (10 s), 69 °C (30 s, decreasing by 1 °C per cycle), 72 °C (1 min); 18 standard cycles at 98 °C (10 s), 51°C (30 s), 72 °C (1 min); final extension at 72 °C (2 min). The product was sequenced bidirectionally by the GeneCreate (Wuhan, China). The sequence was assembled using SeqMan v7.1.0 (DNA Star).

### ﻿Phylogenetic analyses

To determine the phylogenetic position of the new species, the new SSU rDNA sequences, along with those of 67 other hypotrichs retrieved from GenBank database (Fig. 4), were utilized for tree construction. Four euplotids, namely *Diophrysscutum*JF694040, *Uronychiamulticirrus*EU267929, *Apodiophrysovalis*GU477634, and *Paradiophryszhangi*FJ870076, were employed as outgroup taxa. Sequence alignment was performed using the MAFFT v. 7.525 module in PHYLOSUITE v. 1.2.3 (Rozewicki et al. 2019; Zhang et al. 2020; Xiang et al. 2023). Both ends of the alignment were trimmed manually using PHYLOSUITE v. 1.2.3 (Zhang et al. 2020; Xiang et al. 2023) resulting in a final alignment with 1557 sites. Subsequently, a maximum likelihood (ML) analysis with 1000 standard bootstrap pseudoreplicates was conducted using IQ-TREE v. 2.4.0, with the TIM2 + F + R3 model (Minh et al. 2020). Bayesian inference (BI) was carried out using MRBAYES v. 3.2.7a on MODELFINDER v. 2.2.0 employing the GTR + F + I + G4 model (Ronquist et al. 2012; Kalyaanamoorthy et al. 2017). The ITOL v. 7 server was used to visualize the phylogenetic trees (Ivica and Peer 2024).

Phylogenetic support thresholds were categorized as follows: bootstrap values ≥ 95 (strong), 71–94 (moderate), 50–70 (weak), and < 50 (unsupported) following Hillis and Bull (1993). Bayesian nodal confidence was evaluated using posterior probabilities with ≥ 0.95 indicating robust support (Alfaro et al. 2003).

The SSU rDNA sequence of *Oxytrichachongqingica* sp. nov. was aligned with phylogenetically relevant homologs following methods described by Rozewicki et al. (2019). The alignment was curated by removing ambiguously aligned terminal regions and subsequently analyzed in BIOEDIT v. 7.0.5.2 (Hall 1999) to generate pairwise similarity matrices based on global sequence comparisons.

## ﻿Results

### ﻿Taxonomy

**Class Spirotrichea Bütschli, 1889**

**Subclass Hypotrichia Stein, 1859**

**Order Sporadotrichida Fauré-Fremiet, 1961**

**Family Oxytrichidae Ehrenberg, 1838**

**Genus *Oxytricha* Bory de Saint-Vincent in Lamouroux et al., 1824**

#### ﻿ Oxytricha chongqingica sp. nov.

Taxon classificationAnimaliaSporadotrichidaOxytrichidae

69EB1241-A3A6-5670-8B9C-29D8778CF16B

https://zoobank.org/1638B342-9947-4D0E-BAC2-E5FE5F735AC5

**Figure 2.**
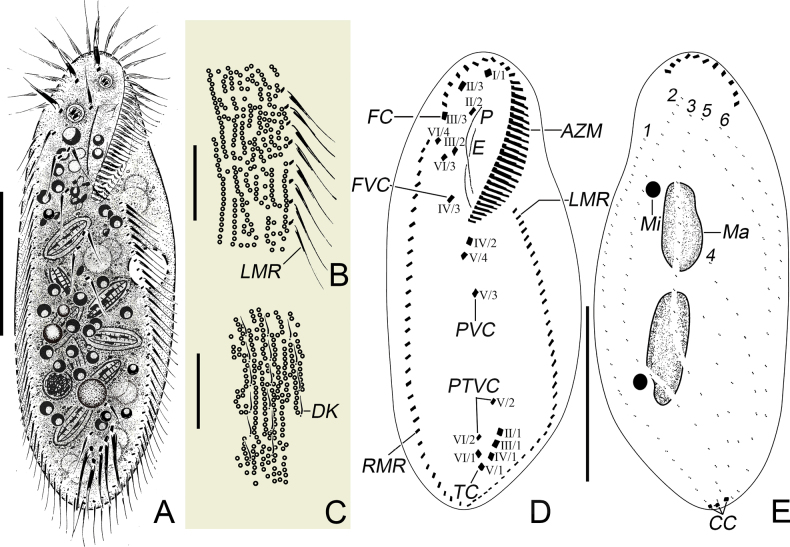
*Oxytrichachongqingica* sp. nov. from life (**A–C**) and after protargol staining (**D**, **E**). **A.** Ventral view of a typical individual; **B, C.** Cortical granules arranged in long rows on ventral (**B**) and dorsal (**C**) side; **D, E.** Ventral (**D**) and dorsal (**E**) view of the holotype specimen. AZM, adoral zone of membranelles; CC, caudal cirri; DK, dorsal kineties; E, endoral; FC, frontal cirri; FVC, frontoventral cirri; LMR, left marginal row; Ma, macronuclear nodule; Mi, micronucleus; P, paroral; PTVC, pretransverse ventral cirri; PVC, postoral ventral cirri; RMR, right marginal row; TC, transverse cirri; 1–6, dorsal kineties 1–6. Scale bars: 50 μm (**A**, **D**, **E**); 15 μm (**B**, **C**).

**Figure 3.**
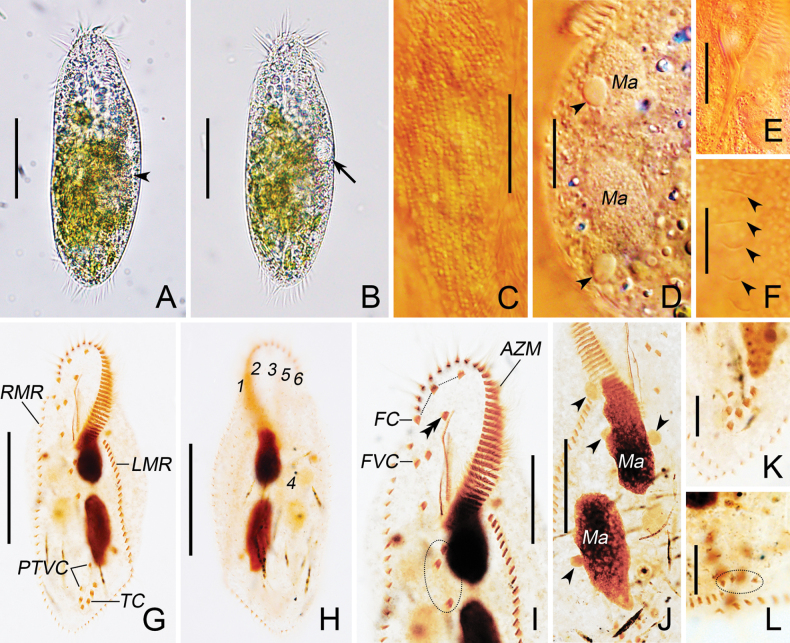
*Oxytrichachongqingica* sp. nov. from life (**A–F**) and after protargol staining (**G–L**). **A, B.** Body in life; arrowhead indicates the collecting canal, and arrow marks the contractile vacuole; **C.** Cortical granules; **D, J.** Macronuclear nodules and micronuclei (arrowheads); **E.** Pharyngeal fibres; **F.** Dorsal cilia (arrowheads); **G, H.** Ventral (**G**) and dorsal (**H**) view of holotype, showing cirral pattern; **I.** Anterior part of ventral surface, showing buccal cirrus (double-arrowhead) and postoral ventral cirri (in ellipse); **K.** Individual with five transverse cirri; **L.** Caudal cirri (in ellipse). AZM, adoral zone of membranelles; FC, frontal cirri; FVC, frontoventral cirri; LMR, left marginal row; Ma, macronuclear nodule; PTVC, pretransverse ventral cirri; RMR, right marginal row; TC, transverse cirri; 1–6, dorsal kineties 1–6. Scale bars: 50 μm (**A, B, G, H**); 20 μm (**C–E, I, J**); 10 μm (**F, K, L**).

Figs 2A–E , 3A–L ; Table 1

##### Diagnosis.

Body size 100–160 × 40–60 μm in vivo, elliptical to elongate-elliptical in outline. Two macronuclear nodules and one to five micronuclei. Contractile vacuole located at mid-body close to left margin. Cortical granules about 1 μm across, arranged in long rows, colorless. The adoral zone occupying 35–44% of the body length, consisting of 37–49 adoral membranelles. Paroral and endoral arranged in a typical *Oxytricha*-pattern. Typical 18 frontoventral-transverse cirri, buccal cirrus positioned at the anterior end of paroral, cirrus III/2 close to and slightly ahead of the level of cirrus VI/3, cirrus V/4 behind cirrus IV/2, the distances between cirri V/3 and V/4 and between cirri V/2 and VI/2 obvious shorter than that between cirri V/3 and V/2. Right and left marginal rows with 32–46 and 33–43 cirri, respectively, confluent at the posterior end of cell. Usually, six dorsal kineties including two dorsomarginal kineties. Dorsal kinety 3 terminating caudally, dorsal kineties 3 and 4 connected by four or five dikinetids, and dorsal kinety 5 ending at approximately 80% down length of body. Three caudal cirri, each one at the posterior end of dorsal kineties 1, 2, and 4.

##### Type material.

The protargol-stained slide (no. XXJ202408130901A) with the holotype specimen (Figs 2D, E, 3G, H) marked with an ink circle, and two paratype slides (no. XXJ202408130901B, C) are deposited in the Laboratory of Protozoological Biodiversity and Evolution in Wetland, Shaanxi Normal University (SNNU), China.

##### Type locality.

Soil from Yintiaoling Nature Reserve in Chongqing, southwestern China (31°31'26"N, 109°49'31"E).

##### Etymology.

The species-group name “*chongqingica*” refers to Chongqing, the type locality.

##### Description.

Body size 100–160 × 40–60 μm (*n* = 7) in vivo, 110–170 × 40–100 μm after protargol staining. Ratio of body length to width 2.5–3.0 in vivo. Flexible but not contractile, elliptical to elongate-elliptical in outline with anterior and posterior ends slightly narrowly rounded. Right margin slightly flat and left margin distinctly convex (Figs 2A, 3A, B; Table 1). Nuclear apparatus consisting of two macronuclear nodules and one to five micronuclei. Macronuclear nodules usually located at 33% and 66% down length of body, respectively, and slightly left of the cell mid-line, about 25 × 15 μm in vivo. Micronuclei attached to macronuclear nodules at variable positions, each about 7 μm across in vivo (Figs 2E, 3D, J; Table 1). Contractile vacuole about 13 μm in diameter when fully expanded, located at about mid-body close to left margin, contracting at intervals of about 10 s, with two non-obvious collecting canals (Figs 2A, 3A, B). Cortical granules arranged in long rows on both sides, colorless, about 1.0 μm across (Figs 2B, C, 3C). Cytoplasm colorless, filled with numerous food vacuoles (2–10 μm in diameter), lipid droplets (0.5–2 μm across), and diatoms (up to 21 μm in length). Movement usually crawling moderately rapidly on the bottom of a petri dish or other substrates, when in suspension, swimming rapidly while spiraling in a counter-clockwise helix.

**Table 1.** Morphometric characterization of *Oxytrichachongqingica* sp. nov.

Character*	H	Min	Max	Mean	Med	SD	CV	*n*
Body, length	128.6	109.2	169.1	140.4	145.3	19.1	13.6	21
Body, width	53.7	43.6	100.9	72.1	71.2	18.1	25.1	21
Body length: width, ratio	2.39	1.55	2.54	2.02	1.92	0.34	16.78	21
Macronuclear nodules, number	2	2	2	2.0	2	0	0	21
Macronuclear nodules, length	25.6	22.0	64.9	38.8	40.1	10.7	27.6	21
Macronuclear nodules, width	11.6	6.1	22.8	12.8	12.3	4.3	34.0	21
Micronuclei, number	2	1	5	2.8	3	1.1	41.1	21
Micronuclei, length	4.3	3.2	6.3	4.9	4.9	0.9	18.6	21
Micronuclei, width	4.3	3.1	6.0	4.0	3.9	0.8	20.2	21
Adoral zone of membranelles, length	48.9	43.3	70.8	56.2	58.1	8.5	15.1	21
Adoral zone of membranelles length, % of body length	38.0	34.9	44.4	39.8	39.5	3.1	7.7	21
Adoral membranelles, number	36	37	49	43.6	43	3.6	8.2	21
DE-value	0.32	0.26	0.37	0.31	0.30	0.03	10.28	21
Cell apex to buccal cirrus, distance	15.5	11.2	22.5	17.7	17.3	2.8	16.1	21
Buccal cirrus, number	1	1	1	1.0	1	0	0	21
Frontal cirri, number	3	3	3	3.0	3	0	0	21
Frontoventral cirri, number	4	4	4	4.0	4	0	0	21
Postoral ventral cirri, number	3	3	3	3.0	3	0	0	21
Pretransverse ventral cirri, number	2	2	2	2.0	2	0	0	21
Transverse cirri, number	5	4	5	4.9	5	0.3	6.1	21
Cirri IV/2 and V/4, distance	4.5	2.8	8.0	4.8	4.5	1.3	27.2	21
Cirri V/3 and V/4, distance	11.2	9.0	15.5	11.6	11.4	1.6	13.7	21
Cirri V/3 and V/2, distance	31.5	24.7	50.2	33.6	31.8	7.3	21.7	21
Cirri in right marginal row, number	32	32	46	39.8	40	3.8	9.5	21
Cirri in left marginal row, number	37	33	43	38.1	39	2.9	7.7	21
Dorsal kineties, number	6	6	7	6.1	6	0.4	5.8	21
Dikinetids in dorsal kinety 1, number	34	33	52	43.1	43	5.2	12.0	21
Dikinetids in dorsal kinety 2, number	29	25	39	33.1	33	3.6	10.8	21
Dikinetids in dorsal kinety 3, number	20	19	29	23.1	23	3.4	14.8	21
Dikinetids in dorsal kinety 4, number	13	9	15	11.0	11	1.7	15.4	21
Dikinetids in dorsal kinety 5, number	17	15	28	21.4	21	3.6	16.8	21
Dikinetids in dorsal kinety 6, number	14	9	19	12.9	13	2.4	18.6	21
Caudal cirri, number	3	3	3	3.0	3	0	0	21

*All data are based on protargol-stained specimens and measurements in μm. Abbreviations: CV, coefficient of variation in %; H, holotype; Max, maximum; Mean, arithmetic mean; Med, median; Min, minimum; *n*, sample size; SD, standard deviation.

Adoral zone occupying 35–44% of body length in protargol preparations and consisting of 37–49 membranelles, and cilia extending up to 21 μm in length (Figs 2A, D, 3G, I; Table 1). DE-value 0.26–0.37 (Table 1). Buccal cavity flat. Endoral and paroral bending and optically intersecting with each other. Pharyngeal fibres inconspicuous after protargol impregnation (Figs 2D, 3E, I).

Three slightly enlarged frontal cirri, with cilia approximately 16 μm long, arranged in an oblique pseudorow, with rightmost cirrus located behind distal end of adoral zone of membranelles. Single buccal cirrus positioned at the anterior end of the paroral. Four frontoventral cirri, characteristically arranged, namely, cirrus III/2 close to and slightly ahead of level of cirrus VI/3. Three postoral ventral cirri, cirrus V/4 positioned behind level of cirrus IV/2, the distances between cirri V/3 and V/4, and between cirri V/2 and VI/2 shorter than that between cirri V/3 and V/2. Two pretransverse ventral cirri, cirrus VI/2 located ahead of rightmost transverse cirrus. Usually, five (rarely four, only two in 21) slightly enlarged transverse cirri arranged in hook-shaped pseudorow, with cilia about 20 μm long in vivo. One right and one left marginal row, with cilia about 15 μm long, consisting of 32–46 and 33–43 cirri, respectively. Right marginal row commencing at level of VI/4, while left marginal row starting near posterior end of adoral zone. Both terminating at posterior end of cell and confluent (Figs 2D, 3G, I, K; Table 1).

Usually six (rarely seven, only four in 21) dorsal kineties including two (rarely three, only four in 21) dorsomarginal kineties, cilia about 4 μm in vivo. Dorsal kinety 1 commencing subapically, while kineties 2 and 3 starting apically, all ending at posterior end of cell, comprising 33–52, 25–39, and 19–29 dikinetids, respectively. Dorsal kinety 4 commencing at about 55% down length of body, terminating at posterior end of cell, and consisting of 9–15 dikinetids. Dorsal kinety 4 not clearly separated from kinety 3, four to six dikinetids existing between them. Dorsal kinety 5 (dorsomarginal kinety) stretching to approximately 80% down length of body and composed of 15–28 dikinetids, while dorsal kinety 6 (dorsomarginal kinety) terminating near midbody and consisting of 9–19 dikinetids. Dorsal kinety 7 (dorsomarginal kinety), if presenting, positioned at about 25% down length of body near right body margin, consisting of one or two dikinetids. Three caudal cirri, each one at the posterior end of dorsal kineties 1, 2, and 4 (Figs 2E, 3H, L; Table 1).

### ﻿Phylogenetic analyses (Fig. 4)

The SSU rDNA sequence of *Oxytrichachongqingica* sp. nov. has been deposited in GenBank with the accession number PV476686. The length and GC content of the novel species are 1672 bp and 45.78%, respectively.

**Figure 4.**
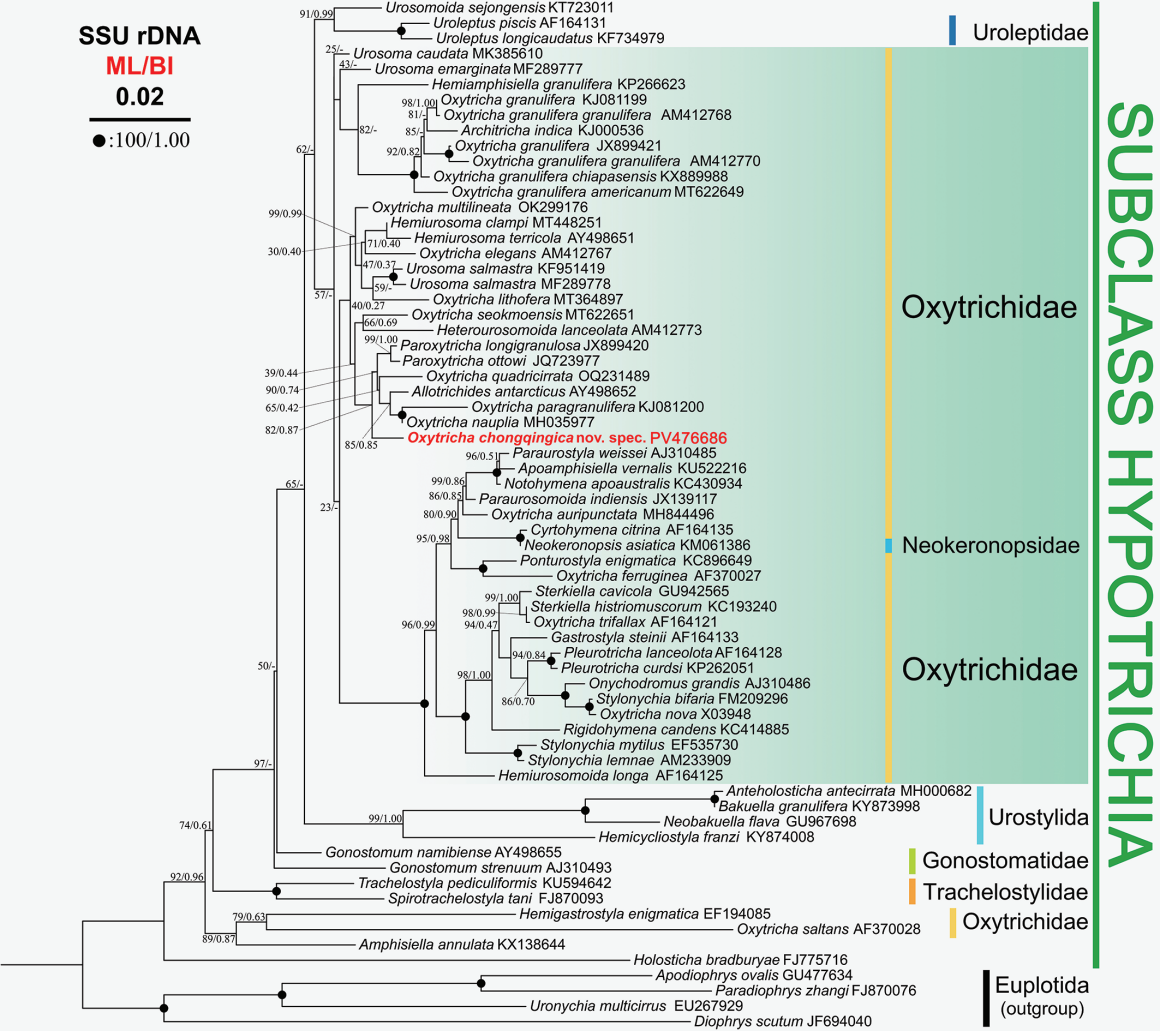
Maximum-likelihood (ML) tree based on 18S rRNA gene sequences, showing the phylogenetic position of *Oxytrichachongqingica* sp. nov. The new sequence is indicated in bold red. Support values for nodes are for ML and BI, respectively (ML/BI). Disagreements in ML and BI tree topologies are indicated by “-”. Fully supported branches are marked with solid circles at the nodes. All branches are drawn to scale. The scale bar corresponds to 0.02 expected substitutions per site.

Phylogenetic trees resulting from maximum likelihood (ML) and Bayesian inference (BI) analyses demonstrate analogous topologies; consequently, solely the ML tree is presented, accompanied by both bootstrap values (ML) and posterior probabilities (BI) (Fig. 4). In the phylogenetic tree, the new species *Oxytrichachongqingica* sp. nov. clusters within the clade containing *O.nauplia* Berger & Foissner, 1987, *Allotrichidesantarcticus* (Berger, 1999) Foissner 2016, *O.paragranulifera*Shao et al., 2014, *O.quadricirrata* Blatterer & Foissner, 1988, *Paroxytrichalongigranulosa* (Berger & Foissner, 1989) Foissner 2016, and *P.ottowi* (Foissner, 1997) Foissner 2016, with support (82 ML and 0.87 BI). These six species were selected as molecularly related taxa of *O.chongqingica* sp. nov. The sequence similarities among *O.chongqingica* sp. nov. and these six species vary from 96.9% (*O.paragranulifera*KJ081200) to 98.7% (*P.longigranulosa*JX899420), with unmatched sites from 19 (*P.longigranulosa*JX899420) to 48 (*O.paragranulifera*KJ081200) (Table 3).

## ﻿Discussion

### ﻿Comparison *Oxytrichachongqingica* sp. nov. with congeners

Based on the body size, shape, ventral and dorsal ciliature, pattern of endoral and paroral and number of macronuclear nodules, *Oxytrichachongqingica* sp. nov. should be compared with the following congeners, namely, *O.aeruginosa* Wrzesniowskiego, 1866, *O.bimembranata* Shibuya, 1929, *O.granulifera* Foissner & Adam, 1983, *O.longicirrata* Kahl, 1932, *O.matritensis* Ramirez-Montesinos & Perez-Silva, 1966, *O.multiseta* Dragesco, 1966, *O.paragranulifera*Shao et al., 2014, *O.procera* Kahl, 1932, *O.proximata* Shibuya, 1930, *O.quadricirrata* Blatterer & Foissner, 1988, *O.seokmoensis* Kim & Min, 2019, and *O.variabilis* Grolière, 1975 (Berger 1999; Shao et al. 2014; Foissner 2016; Méndez‐Sánchez et al. 2018; Kim and Min 2019; Zhu et al. 2021; Bharti and Kumar 2023).

*Oxytrichaaeruginosa* contrasts with *Oxytrichachongqingica* sp. nov. in having black and orange-yellow (vs colorless) cortical granules (Berger 1999).

Compared to *Oxytrichachongqingica* sp. nov., *O.bimembranata* is very different in cortical granules absent (vs. present), and contractile vacuole at level of buccal vertex (vs at about mid-body) (Berger 1999).

Although *O.procera* is a poorly known species, it can still be distinguished from *Oxytrichachongqingica* sp. nov. in cell shape (slender spindle-shaped vs usually elliptical), positions of contractile vacuole (distinctly in front of mid-body vs at about mid-body) and transverse cirri (caudally vs subcaudally), as well as length of cilia of caudal cirri (distinctly longer than cilia of transverse cirri vs. inconspicuous) (Berger 1999).

*Oxytrichagranulifera* is mainly distinguishable from *Oxytrichachongqingica* sp. nov. in dorsal kineties 3 and 4 clearly separated (vs. connected), and dorsal kinety 5 terminates at about mid-body (vs 80% of cell length) of cell. In addition, differences exist in sequence similarities (97.6%–97.8%) and nucleotides 32–38 (Table 2) (Berger 1999; Shao et al. 2014; Méndez‐Sánchez et al. 2018; Zhu et al. 2021).

**Table 2.** Sequence similarities (below diagonal) and nucleotide differences (above diagonal) among *Oxytrichachongqingica* sp. nov. and *O.granulifera*.

Sequences	1	2	3	4	5	6	7	8
1. *Oxytrichachongqingica* sp. nov.		37	34	35	35	38	32	36
2. *O.granulifera*AF164122	0.976		3	18	4	20	12	5
3. *O.granulifera*AF508762, AM412768, AM412769, AM412771, AM412772, MG836525, MG836526, MG836527, MG836528, MG836529, MG836530	0.978	0.998		15	1	17	9	2
4. *O.granulifera*AM412770	0.978	0.988	0.99		16	26	18	17
5. *O.granulifera*KJ081199, MG836531, MG836532, MG836533, MG836534, MG836535, MG836536	0.978	0.997	0.999	0.989		16	8	3
6. *O.granulifera* ssp. MT622649	0.976	0.987	0.989	0.983	0.989		16	19
7. *O.granuliferachiapasensis*KX889988	0.979	0.992	0.994	0.988	0.994	0.989		11
8. *O.granuliferagranulifera*MW143561, MW143562	0.977	0.996	0.998	0.989	0.998	0.988	0.993	

**Table 3.** Sequence similarities (below diagonal) and nucleotide differences (above diagonal) among *Oxytrichachongqingica* sp. nov. and six molecularly related species.

Sequences	1	2	3	4	5	6	7
*Oxytrichachongqingica* sp. nov. PV476686		19	22	25	24	48	22
*Paroxytrichalongigranulosa* JX899420	0.987		7	19	13	35	9
*Paroxytrichaottowi* JQ723977	0.986	0.995		22	18	40	14
*Allotrichidesantarcticus* AY498652	0.984	0.991	0.988		18	46	20
*Oxytrichaquadricirrata* OQ231489	0.984	0.987	0.986	0.988		36	10
*Oxytrichaparagranulifera* KJ081200	0.969	0.977	0.974	0.977	0.97		26
*Oxytrichanauplia* MH035977	0.986	0.994	0.991	0.993	0.987	0.983	

*Oxytrichamultiseta* is different from *Oxytrichachongqingica* sp. nov. in numbers of transverse cirri (six or seven vs four or five), and adoral membranelles (26–29 vs 37–49), as well as ratio of body length: width (about 3.6 vs 2.5–3.0) (Berger 1999).

In comparison with *Oxytrichachongqingica* sp. nov., *Oxytrichamatritensis* manifests distinguishing characteristics in the absence (vs presence) of caudal cirri, and numbers of cirri in right (about 17, data from drawing vs 32–46) and left (about 15, data from drawing vs 33–43) marginal rows (Berger 1999).

*Oxytrichaproximata* can be separated from *Oxytrichachongqingica* sp. nov. by cortical granules absent (vs present), ratio of body length to width about 2.2 (vs 2.5–3.0), and distance between cirri V/2 and V/3 almost equal to (vs largely greater than) those between V/4 and V/3, and V/2 and VI/2 (Berger 1999).

*Oxytrichaparagranulifera* differs from *O.chongqingica* sp. nov. in having *Stylonychia*-patterned (vs *Oxytricha*-patterned) endoral and paroral, as well as lower numbers of adoral membranelles (25–29 vs 37–49), cirri in right (18–25 vs 32–46) and left (18–25 vs 33–43) marginal rows, and dikinetids in kineties 1 (15–23 vs 33–52), 2 (16–23 vs 25–39), 3 (13–17 vs 19–29), 4 (six or seven vs 9–15), 5 (six or seven vs 15–28), and 6 (two vs 9–19) (Shao et al. 2014).

*Oxytrichaquadricirrata* differs from *Oxytrichachongqingica* sp. nov. in a smaller size in life (70–100 × 20–30 μm vs 100–160 × 40–60 μm), lower numbers of adoral membranelles (19–21 vs 37–49), cirri in right (14–17 vs 32–46) and left (13–18 vs 33–43) marginal rows, dikinetids in kineties 1 (9–14 vs 33–52), 2 (7–11 vs 25–39), 3 (5–14 vs 19–29), 4 (three to seven vs 9–15), 5 (three or four vs 15–28) and 6 (one or two vs 9–19), as well as dorsal kinety 5 terminates at 40% (vs 80%) down length of cell (Berger 1999, Bharti and Kumar 2023).

*Oxytrichaseokmoensis* is distinct from *Oxytrichachongqingica* sp. nov. in lower numbers of dikinetids in dorsal kineties 1 (20–26 vs 33–52), 3 (12–17 vs 19–29), and 6 (four to six vs 9–19), as well as ends of right and left marginal rows separate (vs confluent) (Kim and Min 2019).

*Oxytrichavariabilis* can be distinguished from *Oxytrichachongqingica* sp. nov. by numbers of postoral ventral cirri (five vs three) and dorsomarginal kineties (one vs two or three) (Berger 1999).

### ﻿Phylogenetic analyses

The SSU rDNA phylogenetic construction revealed a non-monophyletic group of *Oxytricha* species, consistent with previous molecular phylogenetic studies (Kwon and Shin 2008; Fan et al. 2021; Wang et al. 2021; Jin et al. 2022; Bharti and Kumar 2023). Notably, *Oxytrichachongqingica* sp. nov. formed a weak to moderately supported clade with *O.nauplia*, *Allotrichidesantarcticus*, *O.paragranulifera*, *O.quadricirrata*, *Paroxytrichalongigranulosa*, and *P.ottowi*. This phylogenetic association is supported by sharing morphological characteristics (e.g. 18 frontoventral-transverse cirri, dorsal kinety 3 produces kinety 4 during ontogenesis and 3 caudal cirri produced by dorsal kineties 1, 2, 4 in total) between the novel species and the known species. Interestingly, despite their striking morphological similarities, the SSU rDNA sequence of *O.chongqingica* sp. nov. was found to cluster phylogenetically distant from *O.granulifera*. This result possibly indicates the necessity of incorporating multiple genetic loci, in addition to SSU rDNA, to clarify phylogenetic relationships. Moreover, sequencing molecular data for morphologically defined species is critical for robust evolutionary reconstructions.

## Supplementary Material

XML Treatment for Oxytricha chongqingica

## ﻿Additional information

### Use of AI

This study utilized DeepSeek solely for language polishing and grammatical refinement.

### Author ORCIDs

Haifeng Han https://orcid.org/0009-0009-8151-572X

Zhisheng Zhang https://orcid.org/0000-0002-9304-1789

Chen Shao https://orcid.org/0000-0001-8474-3204

### Data availability

All of the data that support the findings of this study are available in the main text.
